# High Security Settings in Flanders: An Analysis of Discharged and Long-Term Forensic Psychiatric Patients

**DOI:** 10.3389/fpsyt.2022.826406

**Published:** 2022-07-05

**Authors:** Inge Jeandarme, Gokhan Goktas, Jan Boucké, Ingrid Dekkers, Laurent De Boel, Geert Verbeke

**Affiliations:** ^1^FPC Antwerp, Antwerp, Belgium; ^2^Department of Law and Criminology, Catholic University of Leuven, Leuven, Belgium; ^3^FPC Ghent, Ghent, Belgium; ^4^FPCnv, Ghent, Belgium

**Keywords:** high security, internment, forensic psychiatric center, long-term treatment, referrals

## Abstract

**Background:**

Two Forensic Psychiatric Centres (FPC) were implemented the last decade in Flanders in Ghent (2014) and Antwerp (2017). FPCs are forensic institutions for forensic psychiatric patients with a high recidivism risk and a high security need. The objective of FPCs is to create a care process with sufficient flow (from high to lower forms of security), and transitions (from specialized forensic care to regular psychiatric care).

**Aims:**

To examine the characteristics of the high security population in FPCs, treatment length, number of discharges, and discharge locations and to determine the profile of long-term patients within an FPC.

**Methods:**

A retrospective file study of an admission cohort of 654 patients admitted to FPC Ghent or FPC Antwerp was conducted. Sociodemographic, clinical, judicial and risk characteristics were analyzed. Bivariate analyses were used to test the difference between two groups: the group that was discharged to a lower security level vs. the group of long-term patients.

**Results:**

Most patients had psychosis and personality disorders, while comorbidity was also high. Judicial histories were extensive, with many sexual index offenses. During a 6-year follow-up period, the number of referrals back to prison was low. Nearly a third of the population was discharged to a setting with a lower security level. Long-term patients typically presented with more personality disorders, higher psychopathy traits and higher risk scores and were more frequently subjected to coercive measures during treatment.

**Conclusions:**

The Flemish FPC population is characterized by a high proportion of sex offenders as well as a high proportion of personality-disordered patients. It is this last group, and the group with elevated psychopathy traits, who remain for longer than expected and is difficult to resocialize. This study further highlights the need for clear criteria to assess the conditions of these long-term patients in Flanders.

## Introduction

High security institutions are commonplace among international mental health systems and provide specialist care for patients with enduring psychiatric problems in combination with a high risk of further violence. Research indicate that patients in high security settings in western countries were predominantly Caucasian male, between 28 and 38 years old on average (1–8). Judicially, index offenses are presented in a diverse manner across studies, making comparisons difficult. Nevertheless, the majority appeared to be admitted for violent offenses and in one in five cases even life crimes (4, 5). In most studies the prevalence rate of sex offenses was <10% (3, 4, 9), while some studies reported up to 25% of sex offenses (7). Clinically the most common psychiatric diagnoses involved psychotic disorders (3, 4, 6). In Italy, a diagnosis of personality disorder was negatively associated with admission to a high security forensic unit (3). In contrast personality disorders and substance use disorders were frequently found in Dutch high security populations (10). Treatment length was variable: the median hospital stay in high security institutions in England and Wales was 6.9 years (5), and in Norway, it was <1 year (1). Delayed discharge from secure units included poor response to treatment, ongoing safety issues, and lack of suitable step-down facilities (11). Research further indicated other factors related to long-term treatments in secure settings, e.g., psychopathology severity, crime severity, psychotic disorder, history of violence, substance misuse, and non-cooperation with treatment (12–16). After treatment in high security, most discharges (66%) were to an institution with a lower security level; 29% were sent to sheltered housing or outpatient settings, and 5% were referred back to prison (2). In Norway, 35% of referrals were sent back to prison (1). In England and Wales, almost one in five (18.8%) was readmitted to a high security institution after 5 years (5). In Norway, this was the case for one in four patients (1).

Secure forensic services are expensive and highly restrictive; treatment length therefore should be as short as possible and as long as needed. Yet, there are concerns about long-term stays in secure services. What constitutes a ‘long-term' patient is however not clearly defined and differs between countries (17). For example, experts from nine European countries (Italy, Finland, Germany, Ireland, Letland, Poland, Slovenia, Spain, and Switzerland) considered treatment periods between four to 10 years long-term, whereas experts from the Netherlands, England, and Belgium reported that stays of more than 10 years were not unusual (18). In England, one in five patients in high security hospitals had been there for more than 10 years, and a similar proportion had been in medium security for more than 5 years (11). Some if not most of long-term patients are considered treatment resistant and are labeled as ‘longstay' patients. In these patient the shift is less on treatment and more on care and quality of life (19). The Netherlands was the only country with clear criteria to determine longstay status, which can be attributed to patients who have been treated in two separate forensic hospitals for 6 years or more, with no discernible progress (18). In a recent update, the cutoff of 6 years for such status in the Netherlands was abolished (20).

### High Security Forensic Psychiatric Centers Within the Flemish Forensic Care System

Under Belgian law (Act of 5 May 2014, modified by the Potpourri III Act of 4 May 2016), after having committed a crime, people deemed to lack criminal responsibility because of insanity (not guilty by reason of insanity, NGRI) are not punished, but submitted to an internment measure either by investigation or judgment courts. Internment is a security measure with a 2-fold purpose, namely, to protect society and to permit compulsory psychiatric treatment for the forensic patients (further referred to as internees). The Chambers for the Protection of Society (part of the tribunal for the execution of sentences) are responsible for the execution of the internment measure. Treatment referrals by the court are based on the least restrictive measure to protect the public from additional violence, with the highest level of security (Forensic Psychiatric Center; FPC) to the lowest level (community care). In Belgium, treatment can be provided either within a general psychiatric or a forensic psychiatric setting. In Wallonia (southern part of the country), forensic or secure settings have been implemented since 1930. However, in Flanders (northern part of the country), specialized forensic psychiatric care saw a slow start. A prevalence study in September 2004 showed that only 6.7% of Flemish internees were treated in a forensic psychiatric facility (21). Some internees remained in detention for unnecessarily long periods: in December 2013, the average length of detention was 4.8 years, with 14.4% remaining for more than 10 years (22). The European Court of Human Rights (ECHR) criticized the Belgian state for detention of internees in unsuitable facilities, and solicited the government to take structural measures (ECHR 2016, No. 113/2018). In recent decades, the Federal Department of Public Health and Justice and the regional Department of Welfare introduced reforms with a positive impact on expanded forensic care for internees. Among others this resulted in the implementation of two FPCs (FPC Ghent in November 2014, and FPC Antwerp in August 2017) for the group of internees with high security needs and high risk profiles. High security refers to material security (an escape-proof building), procedural security (extensive internal regulations), and relational security (via the Early Recognition Method) (23). Placement in a FPC is mandatory, which implies that neither the FPC nor the internee can refuse placement. Only with severe incidents, unattributed to pathological loss of control, can this realm be initiated by the FPC for a (temporary) return to prison. In other forms of care (e.g., medium security), patients agree to conditions of admission, with institutions using strict inclusion and exclusion criteria. Both the FPC Antwerp (182 beds, with 18 beds for women) and the FPC Ghent (264 beds for male internees) are federal forensic institutions funded partly by the Ministry of Justice (facility services, security, and operational management) and partly by the Ministry of Health (care, medication, and medical fees). FPCs treat internees to reduce new criminal offenses, by removing underlying causes of criminal behavior and rendering them more manageable. As stated, the goal is a responsible return to society: reintegration allows for intermediate forms, from progression to a less secure setting to independent living. Along with the biopsychosocial model (24), the Risk-Need-Responsivity model (25) and the Good Lives Model of rehabilitation (26) are used as theoretical frameworks. Crime analysis and risk assessment in combination with the psychiatric diagnosis form the basis of treatment for all patients. Insight into crime, while dealing with rules and standards, was problematic for internees in the past; for many, treatment in other settings had often gone awry, with safety incidents and rule violations. The ability to deal with boundaries is a necessary treatment objective. At the initial phases of FPC Ghent, average treatment duration was anticipated as 4 years for patients with intellectual disability and 3 years for others, with a large standard deviation. This presumed that long-term treatment settings for treatment resistant patients would soon be available.

### Current Study

Following implementation of high security beds, the current internment policy in Flanders has two objectives. The first is to provide adequate treatment for internees with a high risk and high security profile, avoiding unnecessaraily long detention periods. The second objective is to create a care process with sufficient flow (from high to lower forms of security), and transitions (from specialized forensic care to regular psychiatric care). This study investigates if those objectives were met. The aims of the study are to:

1. Determine sociodemographic, clinical, judicial and risk characteristics of the high security population.
2. Determine treatment length in high security, the number of discharges, and discharge locations.
3. Examine the profile of long-term high security patients.

## Method and Procedure

This study (*N* = 654) includes all current or past admissions to either FPC Ghent or FPC Antwerp in a six-year period, i.e. from the opening of FPC Ghent (17.11.2014) until census date (16.11.2020). Judicial data were obtained via the Central Criminal Register and detention records. Other data were obtained from periodic multidisciplinary reports, submitted by the FPC to the CPS. Demographic, clinical, and risk characteristics were also analyzed.

Only information collected during treatment for clinical or legal purposes was used in this study. The research project was formally approved by the local Ethics Committee of the FPCs. Furthermore, the local ethics committee waived the requirement for ethics approval as approval is not required for studies analyzing anonymized data, in accordance with national legislation (law of 7 may on Experiments on Humans) and institutional requirements.

## Materials

### Sociodemographic Variables

Information on gender, age at first admission, nationality, and residence status was gathered.

### Judicial Variables

For several offenses, the index was classified based on the most serious offense, then clustered into categories: life offenses (murder/manslaughter or attempted murder/manslaughter) > sexual violent offenses (hands-on) > other violent offenses (assault and battery, arson, property crime, threats, or stalking) > other offenses (thefts, and sexual hands-off offenses). The total number of sentences on the record was calculated. A patient was considered a first offender if there were no other convictions and/or internment measures except for the current internment measure.

### Clinical Variables

On a clinical level, previous admissions to a medium-security unit were taken into account. Psychiatric diagnoses were classified in FPC according to either the fourth or fifth edition of the Diagnostic and Statistical Manual of Mental Disorders (DSM). The most recent diagnosis was used. The number of DSM- diagnoses was evaluated. Diagnoses were qualified by the first or primary diagnosis and then clustered into the following categories: personality disorders, psychotic disorders, paraphilic disorders, and other disorders (such as substance-related disorders, or mood disorders). Some diagnoses were calculated irrespective of whether they were established as primary or additional diagnoses: substance misuse, personality disorder, intellectual disability, and paraphilic disorder. Mean intelligence scores were calculated with various testing. The presence of psychopathy was determined on the basis of the Psychopathy Checklist-Revised [PCL-R; (27)]. This score indicates the extent to which psychopathic characteristics were present. The maximum score on the PCL-R is 40, whereas a score of 30 or more is considered by the original author as indicative of psychopathy. In Europe, a score of 25 or more was considered to be indicative of psychopathy (28).

### Risk Profile

Risk profile was defined on the basis of Historical, Clinical and Future - Revision [HKT-R; (29)] The HKT-R is a risk assessment tool used to predict violent and general recidivism. The tool consists of three domains and 33 risk factors: the historical (H) domain (12 risk factors), the clinical (K) domain (14 risk factos), and the future (T) domain (7 risk factors). All risk factors are rated on a 5-point scale, ranging from 0 to 4, where 0 indicates that the indicator is very low risk for the patient, given the circumstances; a score of 4 means there is a high risk. For this study, the numerical score was used to determine the risk level: 0 to 42 = low risk, higher than 42 to 55 = moderate risk, 55 or higher = high risk. In clinical practice the HKT-R is scored every year in order to monitor treatment progress on relevant risk factors. For this study the most recent score was used. The HKT-R was assessed in two possible follow-up situations: either with professional supervision in the FPC and without professional supervision (in society). Scores with more than two missing items were excluded from the analyses (8.3 %, *n* = 54) to ensure that only valid scores would be used. HKT-R assessments were not done by the researchers for the purposes of this study but took place as part of usual care by the clinical team (psychologists in collaboration with criminologists), who had all pursued certified training. According to Fleiss (30) critical values for single measures the interrater reliability for the total HKT-R score was good in previous research (ICC =0.62). Also, according to the classification of Rice and Harris (31), the predictive validity was moderate to large (2 years: AUC =0.78; 5 years: AUC =0.68) (32).

### Treatment

Treatment duration for all patients (admission until census date or discharge date), admitted patients (admission date until census date), and discharged patients (admission date until discharge to a stepdown facility) was analyzed, as well as the place of discharge: stepdown facility (medium security, low security, regular psychiatric service, community care)^1^, prison, or other (e.g., absconding for more than 1 week or death). Place of residence at admission was also determined. For internees admitted directly from prison, the last detention period before admission to the FPC was assessed. During treatment, it was analyzed whether a patient was subjected to coercive measures, as well as the number of coercive measures. Coercive measures concerned seclusion (defined as a placement in a therefore designed, secured room, restricting the patient's freedom to leave it), chemical restraint (referred to medication that is administered against the patient's will, by force or by psychological pressure), and mechanical restraint (defined as applying any external mechanical devices for limiting the patients movement).

## Data Analysis

The data analysis was performed with IBM-SPSS v. 27, Chicago IL, USA. Differences between subpopulations were tested with the Chi-square or Fisher's Exact test in the case of categorical variables, and with the independent *t*-test (normally distributed data) or the Mann-Whitney U test (non-normally distributed data) for continuous variables. The significance level was set at.05. *Post-hoc* comparisons were performed with Bonferroni correction where appropriate. There were missing data with respect to the psychiatric diagnosis (0.6%, *n* = 4), IQ score (26.1%; *n* = 171), PCL-R score (60.9%, *n* = 398), and the HKT-R score (17.7 %; *n* = 116). Valid percentages are provided throughout the text. For comparison analyses, the group of long-term patients (defined as treatment duration of 5 years or longer) was compared to the group of patients discharged to a lower level of security.

## Results

### Descriptive Analyses of Patient Profiles

The FPC population mainly concerned a male population (97.7%, *n* = 639) with Belgian nationality (83.8%, *n* = 548). Moreover, 27 patients (4.1%) were not entitled to stay in Belgium. The mean age at first admission was 42.4 years (*SD* = 12.25, range = 18–77). A small minority was 65 years or older (4.4%, *n* = 29) or 25 years or younger (7.3%, *n* = 48). Intelligence scores were available in 483 files (73.9%) and showed a mean population IQ of 78.7 (*SD* = 17.74, range = 41–140). Nearly half of the population (44.2%, *n* = 289) had been previously treated in a medium security setting prior to admission in the FPC.

On average, internees were subject to 1.7 internment measures (*SD* = 1.26, range = 1–11). The index offenses were life offenses (18.7%, *n* = 122), or sexual violent offenses (26.8%, *n* = 175), along with other violent offenses (42.5%, *n* = 278), or other offenses (12.1%, n = 79). The criminal record included an average of 7.2 convictions or internment measures (*SD* = 7.04, range = 1–45). A minority was regarded as first offenders (15%, *n* = 98).

The primary DSM diagnoses were psychotic disorders (35.7%, *n* = 232), personality disorders (34.8%, *n* = 226), paraphilic disorders (14.0%, *n* = 91) and other disorders (15.5%, *n* = 101). On average, 3.6 DSM diagnoses per internee were classified (*SD* = 1.71, range = 1–10). When all diagnoses were considered, in 63.8% (*n* = 415) there was a personality disorder, 59.8% (*n* = 389) a substance misuse problem, 22.5% showed intellectual disability (*n* = 146), and 23.1% (*n* = 150) showed a paraphilic disorder.

The PCL-R total mean score in the assessed population (39.1%, *n* = 256) was 24.6 (*SD* = 7.39, range = 5.0–37.9). More than half (54.3%, *n* = 139) of the screened population had a PCL-R total score of 25 or greater and a third (32.0%, *n* = 82) had a score of 30 or greater. The mean HKT-R total score was 64.0 (*SD* = 16.63, range = 12.36–106.00) during treatment and 73.0 (*SD* = 16.10, range = 15.45–109.18) during immediate release. The risk of new violent crimes in- and outside the treatment center was estimated as high in the majority of the population (71.4– 86.8%), based on the HKT-R.

### Characteristics of Admissions and Discharges

Most patients were admitted in FPC Ghent (66.5%, *n* = 435), followed by FPC Antwerp (33.5%, *n* = 219). Nearly the entire population (99.2%, *n* = 649) was admitted from prison; the other five internees were transferred from lower security settings. The time in prison prior to FPC admission was 1745.5 days or 4.8 years (*SD* = 2040.73, range = 3–11,212). Over a quarter (28.8%, *n* = 187) stayed in detention for more than 5 years and 14.8% (*n* = 96) for more than 10 years. The mean length of stay for the total population until the census date was 1,033.7 days or 2.8 years (*SD* = 575.59, range 1–2,191).

On the census date (16.11.2020), 393 patients (60.1%) remained in treatment and 261 patients (39.9%) were discharged. Discharged patients were those who completed treatment and were discharged to a lower security level (*n* = 202), and other patients that no longer resided in FPC for other reasons, e.g,. deseased during treatment or sent back to prison. Of the referrals to prison, one was by court decision ex officio, to be subsequently deported to his country of origin (Iraq). A transferal to prison was requested by the FPC due to delayed treatments, combined with continued threatening behavior in two cases, while in the other 13 cases after a serious physically violent incident in which the safety of personnel could no longer be guaranteed. Table 1 shows more details on all discharged patients.

**Table 1:** Discharged patients at census date.

	**Total population** ***N*** **= 654 = 100%**	
	***n***	**%**
**Discharged after treatment completion (*****n*** **= 202)**		
Medium security	108	16.5
Regular medium security unit	70	
Medium security unit for sex offender	21	
Longstay unit	10	
Forensic unit for intellectually disabled persons	7	
Low security	49	7.5
Psychiatric unit	31	
Unit for intellectually disabled persons	18	
Regular psychiatry	23	3.5
Regular psychiatric hospital	12	
Regular unit for intellectually disabled persons	8	
Home for elderly	3	
Community care	22	3.4
Forensic sheltered housing	5	
Regular sheltered housing	3	
Independent living	14	
**Discharged for other reasons (*****n*** **= 59)**		
Transfer other FPC	11	1.7
Time-out	1	0.2
Expelled to country of origin	5	0.8
Released by court	1	0.2
Referred to prison	16	2.4
Absconded for more than seven days	14	2.1
Deceased during treatment	11	1.7
Deceased due to natural cause	8	
Suicide	3	

The mean duration of treatment in the patients discharged to a lower security level was 1,070.4 days or 2.9 years (*SD* = 468.50, range = 39–2,143). Those discharged to a lower security level mainly occurred to residential settings (89.1%) and to a much lesser extent to the community (10.9%). More than half of the 202 discharged patients (53.5%) were sent to a medium security facility. Other patients were referred to a low security facility (24.3%), a general psychiatric facility (11.4%), or a community setting (10.9%) (see Table 1 for details). The mean duration of treatment for patients still in treatment at census date was 1,067.2 days or 2.9 years (*SD* = 617.30 days, range = 1–2,191). During treatment, coercive measures were imposed on half of the population (49.4%). On average this concerned 3.2 coercive measures (*SD* = 7.03, range = 0–74).

### Long-Term Patients

At the census date, 393 patients were in treatment. One fifth of this population (20.4%, *n* = 80/393) were in treatment for more than 5 years. Table 2 summarizes characteristics of the long-term population vs. the discharged group, along with some discernible differences. On the clinical level, there was a difference in patients with a personality disorder [$\chi {{}^{\text{2}}}_{\left(1\right)}$ = 6.49, *p* = 0.01], IQ score (*U* = 4040.50, z = −1.99, *p* = 0.05), and the PCL-R total score (*U* = 747.50, z = −3.80, *p* < 0.001). At the judicial level, the length of prior detention differed (*U* = 5264.50, z = −4.56, *p* < 0.001). In terms of risk assessment, there was a difference in total HKT-R scores [*t*_(200)_ = 5.23, *p* < 0.001 and *t*_(199)_ = 5.69, *p* < 0.001]. More coercive measures were found in the long-term group [$\chi {{}^{\text{2}}}_{\left(1\right)}$ = 17.77, *p* < 0.001] more frequently (*U* = 5483.00, z = −4.75, *p* < 0.001), but there were no differences found in demographic variables.

**Table 2:** Characteristics patients discharged after treatment completion versus long-term patients.

	**Discharged after treatment completion** **(*****n*** **= 202)**		**Long-term treatment patients** **(*****n*** **= 80)**		
	***n*** **(%)**	***M*** **(*SD*)**	***n*** **(%)**	***M*** **(*SD*)**	***p***
**Demographics**					
Belgian nationality	184 (91.1)		72 (90)		0.78
Illegal resident status	0		1 (1.3)		0.28 Fisher
Age at admission (years)		44.6 (12.80)		42.8 (11.29)	0.36 MWU
**Judicial variables**					
Index offense					0.72
Life offenses (or attemps)	37 (18.3)		17 (21.3)		
Sexual violent offenses (hands-on)	64 (31.7)		29 (36.3)		
Other violent offenses	72 (35.6)		25 (31.3)		
Other offenses	29 (14.4)		9 (11.3)		
First offenders	32 (15.8)		6 (7.5)		0.06
Number of convictions on Central Criminal Record		6.0 (5.65)		7.2 (6.31)	0.07 MWU
Duration of detention prior to admission (days)		1858.9 (2143.35)		2656.3 (1965.40)	<0.001** MWU
**Clinical variables**					
IQ score		76.0 (19.12)		80.4 (16.75)	0.05* MWU
Number of DSM-diagnoses		3.6 (1.68)		3.9 (1.89)	0.17 MWU
Substance misuse (comorbidity)	121 (59.9)		46 (57.5)		0.71
Personality disorder (comorbidity)	116 (57.4)		59 (73.8)		0.011*
Intellectual disability (comorbidity)	65 (32.2)		18 (22.5)		0.11
Paraphilic disorder (comorbidity)	59 (29.2)		27 (33.8)		0.46
Psychiatric disorder primary diagnosis					0.18
Psychotic disorder	71 (35.1)		26 (32.5)		
Personality disorder	46 (22.8)		27 (33.8)		
Paraphilic disorder	40 (19.8)		16 (20.0)		
Other disorder	45 (22.3)		11 (13.8)		
PCL-R^∧^ total score		21.9 (7.43)		27.4 (6.42)	<0.001** MWU
**Risk assessment**					
HKT-R^∧∧^ total score “in”		56.2 (15.48)		68.6 (15.75)	<0.001**
HKT-R^∧∧^ total score “out”		65.5 (15.40)		78.6 (14.47)	<0.001**
**Coercive measure**					
Subjected to coercive measure	66 (32.7)		48 (60.0)		<0.001**
Number of coercive measures		1.3 (3.42)		4.6 (7.61)	<0.001** MWU

∧ *PCL-R, Psychopathy Checklist Revised; ^∧∧^HKT-R, Historic, Clinical and Risk Management - Revision*.

*p < 0.05. **p < 0.01.

## Discussion

### Descriptive Analyses of Patient Profiles

The first objective of the present study was to provide a description of the high security population in Flemish FPCs. In terms of age, nationality, gender, average intelligence, and previous admission to a medium security setting, these data were in line with previous research on medium security internees, and largely in line with high security populations in other countries (4, 8, 18, 33). We found some patients with an illegal residence status, constituting a small (4.1%) but problematic group. Apart from a difficult search for a suitable setting in the country of origin it is also almost impossible to transfer such patients to a less secure setting in Flanders, due to residence status and lack of access to social security.

Compared to Flemish medium security populations and most of the high security populations in Italy and England, the high proportion of violent sexual offenses was striking (3, 4). Furthermore, it was remarkable that personality disorders for a primary diagnosis constituted a third of the Flemish high security population, while in Scotland and Italy this was only the case in a minority part of the population (3, 34). In countries such as the Netherlands, where partial responsibility is used, many personality disorders were also found (35). We can only conclude that Flemish psychiatrists-judicial experts - even in a dichotomous system of accountability - are more likely to conclude that these patients were unable to control their behavior. According to De Page and Goethals (36), cultural differences may also play a role in the Belgian context. They compared diagnoses formulated for patients who had been diagnosed by clinicians of both communities and found diagnostic biases for comorbid psychotic and personality disorders. In Wallonia psychotic diagnoses were found more frequently and in Flanders this was the case for personality disorders (33). Multiple diagnoses were actually found, and a high number of substance misuse disorders were part of the current study, which is in line with other research (10, 33).

As expected, the proportion of internees with an increased degree of psychopathy and/or a high recidivism risk was higher than the medium security population (33). Psychopathy and high recidivism risk are vital, as they often form exclusion criteria in settings with lower security levels.

### Characteristics of Admissions and Discharges

The second objective of the study was to provide an overview of admissions, discharges and treatment length. In Flanders, the vast majority of patients were transferred directly from prison to the FPC and referrals cannot be refused. In contrast, almost half of patients admitted in English high security settings were referred by another hospital (4, 6), and admissions can be refused (6). The time spent in prison before FPC admission was extensive, with more than a quarter in detention over 5 years. Compared to high security admissions in for example England, waiting times for admission were considerable [e.g., 0.3 years in (37)].

We anticipate that the current situation will change over the coming years, since the first high security institution only opened in 2014, such that the waiting list was extensive. We already observed a decline in detention periods. During the first 5 years, the last detention period prior to admission to FPC lasted 5.2 years (38), whereas it was 4.8 after 6 years in the current study. In the meantime, clinicians are challenged by this situation. Patients who underwent long detention periods often have attitudes which were adaptive in correctional settings (such as distrust of staff, intimidating behavior, and concealment of symptoms), but which became maladaptive once released (39). In addition, the crime analyses and therapy becomes difficult with a long period between offenses and the start of the therapy. The long waiting time for admission may further explain why the population in FPCs was older compared to those in international studies (4, 6).

The mean length of treatment for the whole group was 2.8 years, which is lower than high security settings in England (5.9 years; 18) and in the Netherlands [8 years in 2017; (40)]. Of course, high security settings in Flanders had been implemented only seven years ago.

During treatment, three patients committed suicide, which is in line with previous research. For example, in the United Kingdom, compared to the general population, the suicide risk was found to be seven times higher in male patients and over 40 times higher in female patients (5).

One of the objectives at the start of the FPCs was to keep referrals to prison as low as possible. Since this concerned only a small minority (2.4% of the total number of patients admitted), this can be considered low compared to other research (1, 2). In Flemish medium security units, nearly one third of patients failed to complete the inpatient forensic treatment programme established to reduce recidivism in violent offenders, even though they were aware of the fact that non-completion would result in a return to prison due to breach of judicial conditions (41). This is worrysome, because non-completion of treatment is related to elevated levels of reoffending, even compared to offenders that were not offered treatment at all (42).

After completed treatments in FPC, the data showed that almost a third of the population (30.9%) could be discharged with a positive recommendation. As is customary in other settings, most discharges were made to a medium security institution. According to Jamieson and Taylor (4), this had more to do with a shortage of settings with lower security and certain clinical preferences, vs. an actual security need: this hypothesis could not be tested in the current study.

### Long-Term Patients

The third objective of the study was to gain more insight into long-term patients. As such, this group was compared to the discharge group, who had already completed treatment.

Treatment length in high security should be as long as needed, but also as short as possible, the goal being a transfer to a less secure setting. However, we identified a group of 80 patients who had remained over 5 years at the census date. At the start of the FPC, treatment duration of 3 to 4 years was anticipated. Six years later, it became clear that this target was not realistic for a subpopulation. Some patients take a longer time to progress, while others will remain too high a risk to be discharged. In other countries (the Netherlands and Germany) such patients are referred to other settings, with less focus on continued treatment and more on care and quality of life in a high security setting. The first high security longstay facility in Flanders will be built in the coming years.

In long-term patients, we found more comorbid personality disorders, higher psychopathy scores, longer detention periods, higher estimated risk of recidivism, and more coercive measures were used. These findings are in line with expectations. Length of stay was associated to seclusion during treatment (43). In the report of the *National Institute for Mental Health*, patients with a personality disorder were often considered untreatable and difficult to manage in both mainstream and forensic care (44). It was questioned whether personality pathology - demanding significant treatment – can be met in secure settings (18). In addition, patients with a high degree of psychopathy are known to make less progress in treatment, causing more incidents and less likely to be resocialized (41, 45). Long-term patients had a higher mean IQ vs. discharged patients in our study, whereas mixed results have been found in the literature for treatment length in intellectually disabled patients (11, 46). Since we did not find a difference with respect to the number of intellectually disabled patients, and more than a quarter of the patients were not tested for IQ, our findings must be interpreted with caution.

### Limitations

One strength of the current study is that the total population in high security settings in Flanders was analyzed. Yet, there still are internees with a high security profile in prison on the waiting list, which indicates that no definitive statements can be made about the entire high security population. Another limitation in the study was inherent to its retrospective nature. It was only possible to rely on information that was already collected in treatment, resulting in missing data. This may have biased results regarding intelligence scores, psychopathy, and risk level. In terms of risk assessment, the amount of missing HKT-R data can be explained by a relatively short hospital stay for a number of patients, and also due to a different risk assessment instrument used for sex offenders.

## Conclusion

In Flanders, there was great need for high security beds over the last few decades, with FPCs filling this link. In our study, we described the profile of admitted patients, determined how their treatment proceeded, and focused on the subgroup of long-term patients. Based on our study, we can conclude that high security internees were those with complex needs, clinically and judicially. The prototypical high security internee is a middle-aged Belgian man, interned after committing a violent crime, having multiple and complex psychiatric problems and a history of serious delinquent behavior. Due to circumstances in Flanders, admission to FPCs occur after a long detention period, making treatment more difficult.

Comparing international forensic psychiatric populations remains difficult. Important differences with regard to the legal system, the organization of forensic psychiatric care, characteristics of local patient groups, and local available treatment facilities all play a key role. Nevertheless, some important differences stand out. The Flemish population is characterized by a high proportion of sex offenders as well as a high proportion of personality-disordered patients. It is this last group, and the group with elevated psychopathy traits, who remain for longer than expected and is difficult to resocialize. The FPCs were established with a goal of resocializing every patient eligible for treatment. After 6 years, treatment was successful for almost one in three internees. However, for another part of the population, resocialization would go less smoothly. In future research, we must distinguish two groups of long-term residents: first, a group who needs long-term treatment, yet is still within the scope of reduced crime risk, enabling transfer at a later stage; second, a group who is treatment-resistant with little prospect of recovery or release while remaining at high risk of reoffending. This last group of so-called longstay patients is difficult to manage in a treatment facility. Many European countries face similar problems despite formal (separate services for longstay patients) or informal care (17). In our view, strict criteria are needed to identify longstay patients, who are best managed in separate longstay institutions that focus on care and quality of life within a restricted environment.

## Data Availability Statement

The raw data supporting the conclusions of this article will be made available by the authors, without undue reservation.

## Author Contributions

IJ, GG, GV, and LD contributed to conception and design of the study. IJ, ID, and JB organized the database. IJ performed the statistical analysis and wrote the first draft of the manuscript. GG, GV, ID, LD, and JB wrote sections of the manuscript. All authors contributed to manuscript revision, read, and approved the submitted version.

## Conflict of Interest

The authors declare that the research was conducted in the absence of any commercial or financial relationships that could be construed as a potential conflict of interest.

## Publisher's Note

All claims expressed in this article are solely those of the authors and do not necessarily represent those of their affiliated organizations, or those of the publisher, the editors and the reviewers. Any product that may be evaluated in this article, or claim that may be made by its manufacturer, is not guaranteed or endorsed by the publisher.

## Footnotes

^1^This classification was made, not based on research, but on structural and procedural security institutions currently used, according to the researchers' estimate, not intended as a definitive classification of security level.

## References

[1] Bjørkly S Sandli C Moger T Stang J. A Follow-up interview of patients 8 years after discharge from a maximum security forensic psychiatry unit in norway. Int J Forensic Ment Health. (2010) 9:343–53. 10.1080/14999013.2010.534698

[2] Buchanan A. Criminal conviction after discharge from special (high security) hospital. Incidence in the first 10 years. Br J Psych. (1998) 172:472–6. 10.1192/bjp.172.6.472 9828985

[3] Capuzzi E Pini E Malerba M Cova F Lax A Mauri S. Factors associated with referrals to high security forensic services among people with severe mental illness and receiving inpatient care in prison. Int J Law Psych. (2019) 62:90–4. 10.1016/j.ijlp.2018.11.003 30616859

[4] Jamieson L Taylor P. Patients leaving english high security hospitals: do discharge cohorts and their progress change over time? Int J Forensic Ment Health. (2005) 4:71–87. 10.1080/14999013.2005.10471214

[5] Jones RM Hales H Butwell M Ferriter M Taylor PJ. Suicide in high security hospital patients. Soc Psychiatry Psychiatr Epidemiol. (2011) 46:723–31. 10.1007/s00127-010-0239-6 20549182

[6] Pimm J Stewart ME Lawrie SM Thomson LD. Detecting the dangerous, violent or criminal patient: an analysis of referrals to maximum security psychiatric care. Med Sci Law. (2004) 44:19–26. 10.1258/rsmmsl.44.1.19 14984211

[7] Berry A Larkin E Taylor P Leese M Watson N Duggan C. Referred to high secure care: determinants of a bed offer/admission and placement after one year. Crim Behav Mental Health. (2003) 13:310–20. 10.1002/cbm.553 14654866

[8] Darjee R Øfstegaard M Thomson L. Schizophrenia in a high-security hospital: longterm forensic, clinical, administrative, and social outcomes. J Forens Psychiatry Psychol. (2017) 28:525–47. 10.1080/14789949.2017.1308537

[9] Sirlier Emir B Kazgan Kiliçaslan A Kurt O Yildiz S. Sociodemographic characteristics of persons treated in the high security forensic psychiatry service: a retrospective study. Medical Records. (2022) 4:73–80. 10.37990/medr.969218

[10] Jankovic M Masthoff E Spreen M de Looff P Bogaerts S. A Latent class analysis of forensic psychiatric patients in relation to risk and protective factors. Front Psychol. (2021) 12:695354. 10.3389/fpsyg.2021.695354 34354640 PMC8329083

[11] Vollm BA Edworthy R Huband N Talbot E Majid S Holley J. Characteristics and pathways of long-stay patients in high and medium secure settings in england; a secondary publication from a large mixed-methods study. Front Psychiatry. (2018) 9:140. 10.3389/fpsyt.2018.00140 29713294 PMC5911489

[12] Dell S Robertson G Parker E. Detention in broadmoor. Factors in length of stay. Br J Psych. (1987) 150:824–7. 10.1192/bjp.150.6.824 3651737

[13] Edwards J Steed P Murray K. Clinical and forensic outcome 2 years and 5 years after admission to a medium secure unit. J Foren Psych Psychol. (2020) 13:68–87. 10.1080/09585180210123294

[14] Huband N Furtado V Schel S Eckert M Cheung N Bulten E. Characteristics and needs of long-stay forensic psychiatric inpatients: a rapid review of the literature. Int J Forensic Ment Health. (2018) 17:45–60. 10.1080/14999013.2017.1405124

[15] Jacques J Spencer S Gilluley P. Long-term care needs in male medium security. Br J Foren Pract. (2010) 12:37–44. 10.5042/bjfp.2010.0424

[16] Shah A Waldron G Boast N Coid J Ullrich S. Factors associated with length of admission at a medium secure forensic psychiatric unit. J Foren Psych Psychol. (2011) 22:496–512. 10.1080/14789949.2011.594902

[17] Sampson S Edworthy R Völlm B Bulten E. Long-term forensic mental health services: an exploratory comparison of 18 European countries. Int J Forensic Ment Health. (2016) 15:1–19. 10.1080/14999013.2016.1221484

[18] Völlm B Edworthy R Holley J Talbot E Majid S Duggan C. A mixed-methods study exploring the characteristics and needs of long-stay patients in high and medium secure settings in England: Implications for service organisation. Health Serv Delivery Res. (2017) 5:5110. 10.3310/hsdr05110 28263522

[19] Holley J Weaver T Vollm B. The experience of long stay in high and medium secure psychiatric hospitals in England: qualitative study of the patient perspective. Int J Ment Health Syst. (2020) 14:25. 10.1186/s13033-020-00358-7 32256688 PMC7104497

[20] Ministerie van Justitie en Veiligheid. (2019). Beleidskader Langdurige Forensisch Psychiatrische Zorg. Available online at: https://www.rijksoverheid.nl/documenten/rapporten/2018/10/08/beleidskader-langdurige-forensisch-psychiatrische-zorg

[21] Cosyns P D'Hont C Janssens D Maes E Verellen R. Geïnterneerden in Belgie. De Cijfers Panopticon. (2007) 1:46–61.

[22] Deckers A Seynnaeve K De Smedt A Dheedene J Vandenplas E Van der Auwera A. Geïnterneerden in Detentie op 24/12/2013. Unpublished Report. (2014).

[23] Kennedy H. Therapeutic uses of security: Mapping forensic mental health services by stratifying risk. Adv Psych Treat. (2002) 8:433–43. 10.1192/apt.8.6.433

[24] Engel GL. The need for a new medical model: a challenge for biomedicine. Science. (1977) 196:129–36. 10.1126/science.847460 847460

[25] Andrews D Bonta J Hoge R. Classification for effective rehabilitation: rediscovering psychology. Psychol Crim Justice Behav. (1990) 17:19–52. 10.1177/0093854890017001004

[26] Ward T Brown M. The good lives model and conceptual issues in offender rehabilitation. Psychol Crime Law. (2004) 10:243–57. 10.1080/10683160410001662744

[27] Hare R. Manual for the Revised Psychopathy Checklist. Toronto, ON: Multi-Health System. (2003).

[28] Cooke DJ Michie C. Psychopathy across cultures: North America and Scotland compared. J Abnorm Psychol. (1999) 108:58–68. 10.1037/0021-843X.108.1.58 10066993

[29] Spreen M Brand E Ter Horst P Bogaerts S. Handleiding en Methodologische Verantwoording HKT-R, Historisch, Klinische en Toekomstige – Revisie. Dr van Mesdag kliniek. (2014).

[30] Fleiss JL. The Design and Analysis of Clinical Experiments. New York, NY: Wiley (1986).

[31] Rice ME Harris GT. Comparing effect sizes in follow-up studies: ROC Area, Cohen's d, and r. Law Hum Behav. (2005) 29:615–20. 10.1007/s10979-005-6832-7 16254746

[32] Bogaerts S Spreen M Ter Horst P Gerlsma C. Predictive validity of the HKT-R Risk assessment tool: two and 5-year violent recidivism in a nationwide sample of dutch forensic psychiatric patients. Int J Offender Ther Comp Criminol. (2018) 62:2259–70. 10.1177/0306624X17717128 28658999 PMC5960839

[33] Jeandarme I. Medium Security Units: Recidivism & Risk Assesment. Tilburg University: Doctoral thesis. (2016).

[34] Crighton J. Defining high, medium, and low security in forensic mental healthcare: the development of the matrix of Security in Scotland. J Foren Psych Psychol. (2009) 20:333–53. 10.1080/14789940802542808

[35] Nagtegaal M Goethals K Meynen G. Tbs-maatregel: kosten en baten in perspectief. Tijdschr Psychiatr. (2016) 58:739–45. 27779292

[36] De Page L Goethals K. Le diagnostic des internés: Y-a-t-il des différences de part et d'autre de la frontière linguistique? Acta Psychiatr Belg. (2019) 118:3–7.

[37] Völlm B Daley A Silva E. Waiting times at a high-security hospital – a survey of the process from referral to admission at Ashworth Hospital. J Forens Psychiatry Psychol. (2009) 20:702–16. 10.1080/14789940903174022

[38] Jeandarme I van Heesch B De Boel L Dekkers I Goktas G Verbeke G. High security geïnterneerden: Wie zijn zij? Waar komen ze vandaan? Waar gaan zij (niet) naartoe? Panopticon. (2020) 41:448–66.

[39] Rotter M Carr WA Magyar M Rosenfeld B. From incarceration to community care: structured assessment of correctional adaptation. J Am Aca Psych Law. (2011) 39:72–7. 21389169

[40] Senn D Bulten E Tomlin J Vollm B. A Comparison of english and dutch long-stay patients in forensic psychiatric care. Front Psychiatry. (2020) 11:574247. 10.3389/fpsyt.2020.574247 33329112 PMC7734324

[41] Jeandarme I Pouls C Oei T Bogaerts S. Forensic psychiatric patients with comorbid psychopathy: double trouble? Int J Foren Mental Health. (2017) 16:149. 10.1080/14999013.2017.1286414

[42] McMurran M Theodosi E. Is treatment non-completion associated with increased reconviction over no treatment? Psychol Crime Law. (2007) 13:333–43. 10.1080/10683160601060374

[43] Davoren M Byrne O O'Connell P O'Neill H O'Reilly K Kennedy H. Factors affecting length of stay in forensic hospital setting: need for therapeutic security and course of admission. BMC Psychiatry. (2015) 15:686. 10.1186/s12888-015-0686-4 26597630 PMC4657210

[44] National Institute for Mental Health in England (2003). Personality disorder: No longer a diagnosis of exclusion. National service framework: Mental Health. Available online at: http://personalitydisorder.org.uk/wp-content/uploads/2015/04/PD-No-longer-a-diagnosis-of-exclusion.pdf

[45] Skeem JL Manchak S Peterson JK. Correctional policy for offenders with mental illness: creating a new paradigm for recidivism reduction. Law Hum Behav. (2011) 35:110–26. 10.1007/s10979-010-9223-7 20390443

[46] Chester V Völlm B Tromans S Kapugama C Alexander R. Long-stay patients with and without intellectual disability in forensic psychiatric settings: comparison of characteristics and needs. BJPsych Open. (2018) 4:226–34. 10.1192/bjo.2018.24 29988967 PMC6034466

